# Exploring Nurses’ Knowledge of and Attitudes Towards the Management of Patients with Heart Failure in Saudi Arabia: A Cross-Sectional Design

**DOI:** 10.3390/healthcare13050522

**Published:** 2025-02-27

**Authors:** Bushra Alshammari, Layla Alanazi, Maha Dardouri, Wiem Aouicha, Mohamed Ayoub Tlili, Sameer A. Alkubati, Awatif Alrasheeday, Ali Mohammed Almuaiqli, Abdulaziz Saeed Alqahtani, Mohammad Saud Alanazi, Azizah Dhaher Alshammri, Fatimah Mansour Alanazi, Najah Sayel Alshammrey, Hajer I. Motakef, Farhan Alshammari

**Affiliations:** 1 Medical Surgical Nursing Department, College of Nursing, University of Hail, Ha’il 2440, Saudi Arabia; 2 Cardiac Ward, King Fahad Specialist Hospital, Tabuk Health Cluster, Tabuk 47717, Saudi Arabia; laila.m.alanazi@gmail.com (L.A.); aalqahtani234@moh.gov.sa (A.S.A.); 3 Department of Maternal and Child Health, College of Nursing, University of Hail, Ha’il 2440, Saudi Arabia; m.dardouri@uoh.edu.sa (M.D.); ha.matekef@uoh.edu.sa (H.I.M.); 4 Faculty of Medicine of Sousse, University of Sousse, Sousse 4003, Tunisia; w.aouicha@uoh.edu.sa (W.A.); mo.tlili@uoh.edu.sa (M.A.T.); 5 Nursing Administration Department, College of Nursing, University of Hail, Ha’il 2440, Saudi Arabia; a.alrasheeday@uoh.edu.sa; 6 Cardiac Catheterization Lap, King Fahad Specialist Hospital, Tabuk Health Cluster, Tabuk 47717, Saudi Arabia; aa.370@hotmail.com; 7 Development and Monitoring Team, Tabuk Health Cluster, Tabuk 47717, Saudi Arabia; mo1403hd@hotmail.com; 8 Emergency Department, King Salman Specialist Hospital, Hail Health Cluster, Ha’il 2440, Saudi Arabia; azdalshammari@moh.gov.sa; 9 Assistant Deputyship for Hospital Services, Ministry of Health, Riyadh 12382, Saudi Arabia; falanazi15@moh.gov.sa; 10 Nursing Executive Administration, Hail Health Cluster, Ha’il 55471, Saudi Arabia; nalshammrey@moh.gov.sa; 11 Department of Pharmaceutics, College of Pharmacy, University of Hail, Ha’il 2440, Saudi Arabia; frh.alshammari@uoh.edu.sa

**Keywords:** heart failure management, nurses’ knowledge, nurses’ attitudes, Saudi Arabia

## Abstract

**Background**: Heart failure (HF) is a significant health burden associated with severe morbidity, mortality, and hospitalization costs and it poses challenges not only to individuals and their families but also to societal and governmental resources. In fact, nurses are indispensable in managing HF patients. The success of patient self-care preparation through education relies not only on the patient’s abilities and attitude but also on the nurse’s proficiency in these tasks and their knowledge and attitudes can significantly impact patient outcomes. This study aimed to evaluate nurses’ knowledge and attitudes regarding HF management in Saudi Arabia. **Methods**: A cross-sectional survey was conducted among a convenience sample of 218 nurses employed at King Fahad Specialist Hospital in Tabuk, Saudi Arabia. Data were collected through an online questionnaire with two sections: one assessing nurses’ knowledge of HF management and the other evaluating their attitudes toward it. The data collection took place between March and June 2024. **Results**: Overall, 55% of nurses showed inadequate knowledge regarding HF management. Further analysis revealed that 46.8% of nurses had a negative attitude towards HF management. Multivariate analysis revealed that graduate nurses (bachelor or diploma) had significantly 4.48 times higher risk to produce inadequate knowledge of HF management in comparison to post-graduate nurses (OR = 4.48; CI 95% [2.18–9.21], *p* < 0.003). Regarding attitudes, nurses who did not receive previous training on HF management had a probability of 2.31 times to produce s negative attitude in comparison to nurses who received training (OR = 2.31; CI 95% [1.33–3.99], *p* = 0.003). **Conclusions**: The study underscores the need for educational programs, continuous professional development, promotion of positive attitudes, and fostering interdisciplinary collaboration to improve HF management. Future research should delve into the long-term impact of interventions, explore organizational factors, and investigate the relationship between knowledge, attitudes, and clinical practices.

## 1. Introduction

Heart failure (HF) is a global health challenge associated with high morbidity, elevated mortality rates, and substantial healthcare costs, impacting millions worldwide [1]. HF is characterized by the heart’s inability to adequately supply blood to meet the body’s needs, which significantly diminishes patients’ quality of life and places a heavy strain on healthcare systems [2,3].

It is estimated that 1.35 million patients are being treated for HF across the Middle East [4]. In Saudi Arabia, the prevalence of HF is notably high, with approximately 32,200 new cases diagnosed each year and an estimated total of 455,222 cases nationally [5]. This growing burden has raised significant concerns within healthcare systems globally, emphasizing the urgent need for effective HF management strategies [6]. HF poses challenges not only to individuals and their families [7] but also to societal and governmental resources [8,9]. The escalating prevalence, coupled with high mortality rates and frequent hospital admissions, leads to substantial economic strains on healthcare systems worldwide [10]. This burden is particularly evident in Saudi Arabia, underscoring the critical need for effective HF management strategies and optimized resource allocation [11,12]. The high health illiteracy among HF patients increases the burden on healthcare providers, requiring strategies to support patients in managing their chronic condition [13,14,15]. Nurses play a crucial role in this effort [16].

In recent decades, the nursing profession has expanded in both roles and responsibilities, with a focus on improving patient outcomes. Nurses play a key role in assessing cardiac deterioration, ensuring treatment adherence, providing education, and offering psychosocial support and counselling. In some settings, they are also authorized to prescribe medications in specific areas [17]. At every stage of the disease, they act as a critical link between patients, their families, and the healthcare team.

In Saudi Arabia, nurses play an increasingly vital role in managing chronic conditions, including HF, as part of national efforts to improve patient outcomes and reduce hospital readmissions [18,19]. According to the Saudi Commission for Health Specialties (SCFHS), nurses are responsible for patient education, symptom monitoring, medication management, and coordination with multidisciplinary teams to optimize care for patients with HF [20,21]. These responsibilities align with Saudi Vision 2030’s emphasis on enhancing healthcare quality and patient-centered care [22]. Despite these advancements, the literature exploring how nurses in Saudi Arabia perceive and implement their roles in HF management remains scarce. Understanding their knowledge and attitudes is essential for developing targeted interventions that enhance patient care and optimize clinical outcomes. Given the significant impact of nurses’ roles on patient care and outcomes [2,23,24], it is essential to enhance their knowledge and attitudes toward HF management [2,25]. Equipping nurses with comprehensive knowledge and fostering a positive approach to HF care can optimize patient care and contribute to improved clinical outcomes [2,26].

While the existing literature on HF in Saudi Arabia offers valuable insights, a notable gap remains in understanding the specific knowledge and attitudes of nurses regarding HF patient management. Investigating the knowledge and attitudes of nurses toward HF management could aid nursing administrators in enhancing patient care by identifying gaps in knowledge, attitudes, and areas for further learning. The current study aims to assess the knowledge and attitudes of nurses regarding the management of patients with HF.

## 2. Materials and Methods

### 2.1. Study Design

In this study, a cross-sectional design was used among nurses at King Fahad specialist hospital in Tabuk, a government hospital providing comprehensive healthcare services, including specialized cardiovascular care. Data were collected between March and June 2024. This study was reported in accordance with the Strengthening the Reporting of Observational Studies in Epidemiology (STROBE) checklist to ensure comprehensive and transparent reporting of the research methods and findings [27].

### 2.2. Sampling and Sample Size

The study recruited a convenience sample of registered nurses at King Fahad Specialist Hospital in Tabuk who met the following inclusion criteria: providing direct care to HF patients, having at least one year of experience, and consenting to participate. Administrators and other non-caregiving staff were excluded.

A total of approximately 500 nurses work in King Fahad specialist hospital. Using Raosoft software (Raosoft, Inc., Seattle, WA, USA), a sample size of 218 nurses was calculated to ensure statistically reliable results. This sample size, based on a 50% response distribution, a 95% confidence level, and a 5% margin of error, provides a robust representation of nurses’ knowledge of and attitudes toward heart failure management [28].

### 2.3. Data Collection Instruments

Data collection was performed using an online questionnaire on Google Forms. The questionnaire link was initially sent to the head of nursing at the hospital, who identified eligible nurses based on the study’s inclusion criteria. The link was then distributed to all eligible nurses via email. The questionnaire included information about the study’s purpose, participation requirements, and the researchers’ contact information for any inquiries. Nurses were informed that returning the completed questionnaire implied their consent to participate. To improve the response rate, reminder emails were sent one week after the initial distribution and again after two weeks.

The questionnaire is composed of three parts. The first part included demographic data such as gender, age, marital status, level of education, job position, years of experience, training course received in HF management, and monthly income.

The second part consisted of nurses’ knowledge of management of patients with HF, developed by Albert et al. [29]. It measures the nurses’ knowledge of management of patients with HF, with 20 items. Response format for the instrument consists of “Yes”, “No”, and “I did not know”. Fifteen items are correct with a response of “false” and five items are correct with a response of “true”. The total score is calculated by summing the total number of items answered correctly. Possible score range is from 0–20 with higher scores indicating greater knowledge. For scoring purposes, correct answers are assigned a point value of “1”, whereas incorrect and “don’t know” responses are scored as “zero”. Knowledge levels are categorized as follows: scores of 0–10 indicate inadequate knowledge, while scores of 11 or above are considered adequate, with 11–15 signifying satisfactory knowledge, 16–18 representing good knowledge, and 19–20 denoting very good knowledge [30]. The correct answers in the knowledge assessment section were determined according to the guidelines referenced by Albert et al. (2002) [29], including those from the American Heart Association (AHA) and the Agency for Healthcare Research and Quality (AHRQ). The questionnaire’s validity and reliability were carefully established. Content and face validity were confirmed by expert HF nurses and patient education specialists [29]. Reliability was ensured through consistent scoring by qualified registered nurses, achieving full agreement on survey items, affirming the tool’s robustness and accuracy [29].

The third part of the questionnaire reported the nurses’ attitudes towards the management of patients with HF developed by Sanad [31]. The instrument is composed of 18 items designed to probe nurses’ perspectives on managing HF patients. Responses were gathered using a 5-point Likert scale, where 1 represented “strongly disagree” and 5 was “strongly agree”. This scale was reversed in three negative questions (1 = strongly agree; 5 = strongly disagree). The scores of the items were summed up and averaged by dividing by the number of items. According to Sanad (2017) [31], the questionnaire’s validity and reliability have been rigorously evaluated and a panel of cardiology experts in both medical and nursing fields assessed the questionnaire for face and content validity, implementing necessary modifications during the development phase. Additionally, the attitude scale’s reliability was tested through a pilot study with 20 nurses, showing strong internal consistency with a Cronbach’s alpha of 0.798. The tool was then finalized based on these results, confirming its validity and reliability [31].

### 2.4. Data Analysis

Data were analyzed using IBM SPSS Statistics version 27. Continuous variables were described in means and standard deviations and categorical variables were presented in frequencies or percentages. The total mean of attitude was calculated and categorized as either positive (from 3.01 to 5) or negative (from 1 to 3). To test the association between dependent variables (knowledge and attitude) and independent variables (gender, age, marital status, level of education, job position, years of experience, if received training course in heart failure management, and monthly income), the Chi Square test was performed. To meet the application conditions of this statistical test, some independent variables were transformed into binary variables by grouping the categories as follows: age (0: <30 years; 1: ≥30 years), level of education (0: graduate (bachelor and diploma); 1: post-graduate (master)), experience (0: <3 years; 1: ≥3 years); income (0: <10,000 Saudi Riyal (SAR); 1: ≥10,000 SAR, noting that 1 USD ≈ 3.75 SAR).

To determine predictive factors of knowledge and attitude, binary logistic regression was performed. The variables entered in the regression model had a *p* < 0.2 in the univariable analysis. The level of statistical significance was at *p* < 0.05 with a confidence interval of 95%.

### 2.5. Ethical Considerations

Ethical approval was obtained from the Ethical Committee at the University of Ha’il (approval number H-2024-099, approved on 4 March 2024) and from the Ethics and Research Committee at King Fahad Specialist Hospital (approval number KFS-2024-293, approved on 10 February 2024). Participation in the study was entirely voluntary, and no identifying information about the nurses, such as their names, was requested.

The initial page of the online survey instrument included comprehensive information about the study. This allowed nurses to read the information provided before beginning the survey and decide whether they were interested in participating. The participants’ acceptance and completion of the survey were consequently taken as their indicated informed consent.

## 3. Results

### Population Characteristics

The survey included 218 nurses, with approximately equal distribution of male (51.4%) and female (48.6%) nurses. More than one-third of nurses were aged between 24 and 29 (38.5%). Regarding nursing training, almost half of the sample (44%) received training in HF management. Income levels were mostly in the 5000–10,000 SAR range (52.8%) as shown in Table 1.

Table 2 shows the rate of correct answers. The question about whether patients with HF should decrease activity and avoid most forms of active exercise received the highest number of correct responses (89.9%). Similarly, there was a strong awareness that coughing and nausea/poor appetite are common symptoms of advanced HF (79.8% correct).

Overall, 55.5% of nurses showed inadequate knowledge and 44.5% showed adequate knowledge (Table 3). The attitude total score was 3.15 ± 0.6. Considering the attitude classification used in this research, the data showed that 46.8% had a negative attitude towards HF management and 53.2% had a positive attitude.

Table 4 shows the factors associated with knowledge and attitude. There were significant differences between knowledge and gender (*p* = 0.003) as well as level of education (*p* < 0.001). In fact, the highest rate of inadequate knowledge was significantly reported in male nurses (73%). Almost half of the nurses (49.5%) who were graduate nurses (bachelor or diploma) reported significantly inadequate knowledge. Regarding attitude, the data showed that the age (*p* = 0.03), the level of education (*p* = 0.03), and previous training in HF management (*p* = 0.002) were significantly associated with nurses’ attitudes.

Table 5 presents the final binary logistic regression models. According to the current findings, graduate nurses (bachelor or diploma) had significantly 4.48 times higher risk to produce inadequate knowledge of HF management in comparison to post-graduate nurses (OR = 4.48; CI 95% [2.18–9.21], *p* = 10^−3^). Female gender reduced the probability of inadequate knowledge in nurses (OR = 0.46; CI 95% [0.26–0.82], *p* = 0.009). Regarding the attitude, nurses who did not receive previous training on HF management had a probability of 2.31 times to produce negative attitude in comparison to nurses who received training (OR = 2.31; CI 95% [1.33–3.99], *p* = 0.003).

## 4. Discussion

HF presents a significant global health challenge, characterized by high morbidity, mortality rates, and substantial healthcare costs [1]. Nurses play a crucial role in HF management, encompassing patient assessment, treatment adherence, education, psychosocial support, and, in some settings, medication prescription [23,32]. Improving nurses’ knowledge and attitudes is therefore essential for optimizing patient outcomes [26].

Previous research has explored nurses’ knowledge and attitudes regarding HF management in various settings. Studies have highlighted knowledge deficits in specific areas, such as patient education [33,34]. However, a significant gap exists in our understanding of nurses’ knowledge of and attitudes toward HF management specifically within the Saudi Arabian context. The existing literature offers valuable insights into the global HF burden and the importance of nurses’ roles, but there is a paucity of research focusing on the knowledge and attitudes of Saudi Arabian nurses. This lack of context-specific data hinders the development of targeted interventions to improve HF care in Saudi Arabia. This study addressed this gap by assessing the knowledge and attitudes of nurses regarding HF patient management at King Fahad Specialist Hospital in Tabuk, Saudi Arabia. By identifying knowledge deficits and influencing factors, this research can inform the development of culturally relevant educational programs and professional development initiatives to enhance the quality of HF care in the region.

### 4.1. Nurses’ Knowledge of HF Management

The findings of this study shed light on the current state of nurses’ knowledge regarding HF management revealing that a majority of nurses, comprising 55.5%, have been assessed as possessing inadequate knowledge regarding the management of patients with HF. The prevalence of inadequate knowledge among a majority of nurses underscores potential gaps in nursing education and training programs. These knowledge deficiencies, particularly in critical areas like dietary considerations, fluid management, and symptom recognition, raise concerns regarding the quality of care delivered to HF patients.

The parallels observed with previous studies by Wang et al. [34] and Ghani et al. [33] may emphasize the global nature of these challenges, suggesting systemic issues within nursing education and professional development. The identification of specific areas of misconception, such as fluid intake recommendations, highlights the need for targeted educational interventions to address these misconceptions and improve the overall competency of nursing staff in managing HF.

However, amidst these challenges, the presence of a subset of nurses demonstrating good to very good knowledge levels signify a potential pathway for improvement. The association between higher educational attainment and specialized training with better knowledge scores, as elucidated by Demissie et al. [2], underscores the importance of investing in advanced education and training programs tailored to HF management.

Indeed, the analysis reveals a significant association between nurses’ graduation level and their knowledge of HF management. Specifically, graduate nurses (holding a bachelor’s or diploma degree) demonstrate a substantially higher risk of inadequate knowledge compared to post-graduate nurses. Post-graduate programs likely offer more comprehensive training in complex medical conditions like HF, enabling nurses to provide higher quality care to patients. Healthcare institutions should consider investing in advanced education opportunities for nursing staff to address knowledge gaps and enhance the standard of care for HF patients [33].

The analysis also identifies gender as a significant factor influencing nurses’ knowledge of HF management. Female nurses exhibit a reduced probability of inadequate knowledge compared to their male counterparts. Actually, this disparity between males and females in the nursing profession in terms of knowledge, attitudes, and practices has been the subject of several studies [35,36,37]. For instance, the article by Woo et al. [36] “Understanding the gender gap in advanced practice nursing” revealed that the advanced practice nurses career-track is unpopular among men where, interestingly, though the odds were seemingly in their favor, male nurses were noted to lack interest in becoming advanced practice nurses. While the reasons behind this gender disparity may not be easily explored, possible explanations could include differences in educational experiences, career trajectories, or access to professional development opportunities. Understanding these gender-based differences can inform targeted interventions aimed at improving knowledge dissemination and educational support for all nurses, irrespective of gender.

### 4.2. Nurses’ Attitudes Towards Patients with HF

The generally favorable attitudes towards HF patient management observed in the present study, particularly concerning the significance of health education and the impact of nursing care, resonate with the findings of Singh et al. [38] and Demissie et al. [2]. These studies collectively underscore nurses’ acknowledgment of their pivotal role in patient education and the management of chronic conditions such as HF. However, the current study also identified areas of negative sentiment, such as the perception that explaining nursing procedures to patients is laborious and the belief that intensive care placement adversely affects patient mental well-being.

The mixed attitudes observed among nurses towards HF patient management reflect a complex interplay of factors shaping nursing practice and patient care delivery. While the generally positive attitudes towards the importance of health education and nursing care highlight nurses’ commitment to patient well-being, the identification of negative sentiments towards certain aspects of patient care underscores underlying challenges and areas for improvement.

Moreover, the significant variations in attitudes based on demographic and professional variables highlight the multifactorial nature of attitudes towards patient care.

The study highlights the critical role of previous training. Nurses who have not received prior training on HF management are more likely to harbor negative attitudes compared to those who have undergone training. The positive correlation between higher educational levels and more favorable attitudes, as demonstrated also by Bit-Lian et al. [39], suggests that investing in advanced education and training programs can not only enhance nurses’ knowledge but also shape their attitudes towards patient care and give them confidence to approach patient care with a positive and proactive mindset, ultimately improving patient outcomes and satisfaction.

Overall, the results of the predictive factors underscore the complex interplay of education, gender, and training in shaping nurses’ knowledge of and attitudes towards HF management. By addressing these factors through targeted interventions, supportive policies, and commitment to excellence in nursing practice, healthcare institutions can strive towards improved patient outcomes and promote professional growth and satisfaction among nursing staff, ultimately enhancing the quality and effectiveness of nursing practice in HF management.

This study presents certain limitations that may influence the interpretation and generalizability of its findings. First, as the data were derived from a single hospital, the results may not be generalizable to other healthcare settings or regions. Secondly, the reliance on self-reported data introduces the potential for response bias, as nurses may consult external sources—such as discussing questions with colleagues, referencing materials, searching online, or sharing answers with others—while completing the questionnaire. This could impact the accuracy of their individual responses and potentially compromise the data’s reliability. Finally, the study’s scope did not include an analysis of organizational factors such as workload, staffing, institutional policies that could significantly influence nurses’ knowledge of and attitudes toward HF. Future research should consider these contextual factors for a more comprehensive view of nurses’ preparedness in managing HF.

## 5. Conclusions

The findings from this study reveal significant gaps in nurses’ knowledge of and attitudes towards HF management, particularly among nurses with lower educational attainment and those lacking specific training in HF care. These results underscore the critical need for targeted educational programs and continuous professional development initiatives to equip nurses with the necessary skills and positive attitudes for managing HF effectively. Enhancing nurses’ knowledge and attitudes is essential to improving patient outcomes, as nurses play a pivotal role in patient education, symptom monitoring, and treatment adherence. The study highlights the importance of prioritizing nurse training in HF management as an integral part of healthcare improvement strategies. Future research should investigate the long-term impact of these educational interventions on patient outcomes and explore organizational factors that may further support effective HF management in diverse healthcare settings.

## Figures and Tables

**Table 1:** Demographic and general data of nurses (N = 218).

Variables	Categories	n	%
Gender	Female	106	48.6
	Male	112	51.4
Age	24–29 years	84	38.5
	30–34 years	72	33.0
	35–39 years	34	15.6
	40–44 years	23	10.6
	More than 45 years	5	2.3
Marital status	Single	119	54.6
	Married	99	45.4
Level of education	Diploma	33	15.1
	Bachelor degree	137	62.9
	Master’s degree	48	22.0
Job Position	Staff nurse	174	79.8
	Manager nurse	44	20.2
Years of experience	Less than 3 years	52	23.9
	4–7 years	94	43.1
	8–15 years	63	28.9
	16 years and more	9	4.1
Received training course in heart failure management	No	122	56.0
	Yes	96	44.0
Monthly Income	Less than 5000	13	6.0
	5000–10,000	115	52.8
	10,000–15,000	81	37.1
	More than 15,000	9	4.1

Nurses’ knowledge of and attitude of nurses towards HF management.

**Table 2:** Nurses’ knowledge of heart failure management (N = 218).

Items	Correct Answer (%)
Fluid Management Awareness	50.5
Dietary Choices	56.4
Dietary Misconceptions	55.5
Recognition of Advanced HF Symptoms	20.2
Physical Activity Perceptions	10.1
Weight Monitoring Practices	50.9
Identifying Fluid Retention	15.6
Medication and Lifestyle Adherence	45.9
Pain Management Misunderstandings	49.5
Shortness of Breath and Sleeping Habits	46.8
Breathlessness During the Night	43.6
Daily Weight Importance Post-Symptom Improvement	47.2
Weight Assessment Practices	56.0
Importance of Daily Weighing in HF Management	45.0
Healthy Dietary Patterns	54.6
Medication Timing Practices	54.1
Blood Pressure and Symptom Reporting	55.5
Reporting Postural Dizziness in HF	52.3
Recognizing and Reporting New or Worsening Fatigue in HF	59.6
Identifying and Reporting Leg Weakness and Reduced Exercise Tolerance in HF	49.5

**Table 3:** Classification of nurses’ knowledge of and attitude towards heart failure management in percentages (N = 218).

Dependent Variables	Categories	%
Knowledge	Inadequate	55.5
	Satisfactory	34.4
	Good	3.2
	Very good	6.9
Attitudes	Positive	53.2
	Negative	46.8

Factors influencing nurses’ knowledge of and attitudes towards HF management.

**Table 4:** Differences in nurses’ knowledge of and attitudes towards heart failure management based on sociodemographic variables.

Variables	Categories	Knowledge (N = 218)			Attitude (N = 218)		
		Adequate	Inadequate	*p*	Positive	Negative	*p*
Age	Less than 30 years	31 (33.0)	53 (44.5)	0.080	31 (31.6)	53 (46.1)	0.032
	30 years or more	63 (67.0)	66 (55.5)		67 (68.4)	62 (53.9)	
Gender	Female	58 (59.8)	48 (39.7)	0.003 *	53 (52.0)	53 (45.7)	0.301
	Male	39 (40.2)	73 (60.3)		49 (48.0)	63 (54.3)	
Marital status	Single	46 (47.4)	73 (60.3)	0.050	55 (53.9)	64 (55.2)	0.802
	Married	51 (52.6)	48 (39.7)		47 (46.1)	52 (44.8)	
Level of education	Graduate	62 (63.9)	108 (89.3)	0.001 *	73 (71.6)	97 (83.6)	0.030 *
	Post-graduate	35 (36.1)	13 (10.7)		29 (28.4)	19 (16.4)	
Years of experience	Less than 3 years	20 (20.6)	32 (26.4)	0.303	22 (21.6)	30 (25.9)	0.402
	3 years or more	70 (79.4)	89 (73.6)		80 (78.4)	86 (74.1)	
Job Position	Staff nurse	77 (79.4)	97 (80.2)	0.801	83 (81.4)	91 (78.4)	0.510
	Manager nurse	20 (20.6)	24 (19.8)		19 (18.6)	25 (21.6)	
Previous training	Yes	44 (45.4)	52 (43.0)	0.703	56 (54.9)	40 (34.5)	0.002 *
	No	53 (54.6)	69 (57.0)		46 (45.1)	76 (65.5)	
Income	Less than 10,000 SAR	55 (58.7)	73 (60.3)	0.501	56 (54.9)	72 (62.1)	0.213
	10,000 SAR or more	42 (41.3)	48 (39.7)		46 (45.1)	44 (37.9)	

* Significant level *p* < 0.05.

**Table 5:** Final binary logistic regression models for knowledge and attitude variables.

Variables.	β	Adjusted OR	CI 95%	SE	*p*
Knowledge					
Gender (Female)	−0.75	0.46	[0.26–0.82]	0.29	0.009
Education (Graduate)	1.5	4.48	[2.18–9.21]	0.36	0.001
Attitude					
Previous training (No)	0.83	2.31	[1.33–3.99]	0.27	0.003

## Data Availability

The data presented in this study are available on request from the corresponding author.

## Abbreviation

The following abbreviation is used in this manuscript:

HF	Heart failure
